# Functional characterization of spectral tuning mechanisms in the great bowerbird short-wavelength sensitive visual pigment (SWS1), and the origins of UV/violet vision in passerines and parrots

**DOI:** 10.1186/1471-2148-13-250

**Published:** 2013-11-13

**Authors:** Ilke van Hazel, Amir Sabouhanian, Lainy Day, John A Endler, Belinda SW Chang

**Affiliations:** 1 Department of Ecology & Evolutionary, Biology University of Toronto, Toronto, Canada; 2 Department of Biology, University of Mississippi, Oxford, Mississippi, USA; 3 Centre for Integrative Ecology, Deakin University, Melbourne, Australia; 4 Department of Cell & Systems Biology, University of Toronto, Toronto, Canada; 5 Centre for the Analysis of Genomes and Evolution, University of Toronto, Toronto, Canada

**Keywords:** Opsins, Ultraviolet, Bird vision, Visual pigment evolution

## Abstract

**Background:**

One of the most striking features of avian vision is the variation in spectral sensitivity of the short wavelength sensitive (SWS1) opsins, which can be divided into two sub-types: violet- and UV- sensitive (VS & UVS). In birds, UVS has been found in both passerines and parrots, groups that were recently shown to be sister orders. While all parrots are thought to be UVS, recent evidence suggests some passerine lineages may also be VS. The great bowerbird (*Chlamydera nuchalis*) is a passerine notable for its courtship behaviours in which males build and decorate elaborate bower structures.

**Results:**

The great bowerbird SWS1 sequence possesses an unusual residue combination at known spectral tuning sites that has not been previously investigated in mutagenesis experiments. In this study, the SWS1 opsin of *C. nuchalis* was expressed along with a series of spectral tuning mutants and ancestral passerine SWS1 pigments, allowing us to investigate spectral tuning mechanisms and explore the evolution of UV/violet sensitivity in early passerines and parrots. The expressed *C. nuchalis* SWS1 opsin was found to be a VS pigment, with a λ_max_ of 403 nm. Bowerbird SWS1 mutants C86F, S90C, and C86S/S90C all shifted λ_max_ into the UV, whereas C86S had no effect. Experimentally recreated ancestral passerine and parrot/passerine SWS1 pigments were both found to be VS, indicating that UV sensitivity evolved independently in passerines and parrots from a VS ancestor.

**Conclusions:**

Our mutagenesis studies indicate that spectral tuning in *C. nuchalis* is mediated by mechanisms similar to those of other birds. Interestingly, our ancestral sequence reconstructions of SWS1 in landbird evolution suggest multiple transitions from VS to UVS, but no instances of the reverse. Our results not only provide a more precise prediction of where these spectral sensitivity shifts occurred, but also confirm the hypothesis that birds are an unusual exception among vertebrates where some descendants re-evolved UVS from a violet type ancestor. The re-evolution of UVS from a VS type pigment has not previously been predicted elsewhere in the vertebrate phylogeny.

## Background

Bowerbirds are a remarkable group of passerine birds in which males build elaborate structures of plant material adorned with coloured objects to attract females. These displays are among the most striking examples of sexually selected traits. Consequently, bowerbirds have become a model system in visual ecology and evolutionary biology, particularly with respect to the evolution of visual signals [1-6]. Birds have a visual system well suited for colour detection with four types of cone visual pigments that span a wide range of the electromagnetic spectrum extending into the ultraviolet (UV). UV based signals in particular can play important roles in avian behaviours [7-9], especially in mate choice in passerines [10,11] and parrots [12].

The first step in vision is the absorption of light by visual pigments in the photoreceptor cells of the retina. Visual pigments consist of an opsin protein covalently bound to a light sensitive chromophore via a Schiff base (SB) link. Absorption of a photon of light triggers a *cis-trans* isomerization in the chromophore that induces subsequent conformational changes in the opsin protein. This change allows the visual pigment to bind and activate the downstream heterotrimeric G-protein, transducin, thus initiating the visual transduction cascade in the photoreceptor cell [13]. The wavelength of maximal absorbance of a visual pigment (λ_max_) is determined by the interactions between the opsin protein and its chromophore, via a process known as spectral tuning [14].

The short-wavelength-sensitive (SWS1) pigments mediate sensitivity to light in the violet to UV range. This group of pigments exhibits the broadest range in spectral sensitivity across vertebrates, and are generally divided into two groups based on λ_max_: violet-sensitive (VS: λ_max_ 388–435 nm) and UV-sensitive (UVS: λ_max_ 355–380 nm) [15]. In SWS1 pigments, spectral tuning mechanisms can be quite complicated, and can differ across vertebrate pigments [16-23]. However, among vertebrates, SWS1 spectral tuning mechanisms in birds appear to be fairly unique and unusually straightforward. Mutagenesis studies in a variety of birds indicate the most important site is 90, with mutations at this site responsible for determining whether a pigment absorbs maximally in the violet or UV [17,18,21,24]. Phenylalanine (F) at site 86 appears to be a second mechanism by which birds achieve UVS because it is found in the SWS1 genes of some birds [25-27], and site-directed mutagenesis studies indicate that it can blue shift wavelength sensitivity in some avian VS-type SWS1 pigments [28] as well as in other vertebrates [16,19,29,30], with the exception of some primates [23]. However, the paucity of mutagenesis studies on SWS1 pigments throughout the diverse avian orders somewhat limits our abilities to extrapolate upon the roles of spectral tuning sites across all birds.

Here, we use site-directed mutagenesis and ancestral reconstruction methods in order to characterize the absorption spectra of ancestral passerine/parrot SWS1 pigments, and to investigate SWS1 spectral tuning mechanisms using the great bowerbird pigment as a model system. Until recently, the parrots and passerines were thought to be divergent orders within landbirds, but in fact have been found to be sister groups in a number of recent studies [31-33], though this relationship is not always recovered [34,35]. The relationship between passerines and parrots is relevant to understanding the evolution of UV/violet vision in birds because both groups are thought to contain UVS due to the presence of C90 [17,36-38], raising the question of when UV sensitivity may have arisen in these groups. Recent results indicate some basal songbird lineages may have VS pigments [39,40] and in fact, a variety of other basal passerine lineages including some flycatchers have also been found to possess S90, suggestive of VS pigments [41]. As one of the basal passerine lineages whose ecology and behaviour have been the subject of detailed study, the great bowerbird (*Chlamydera nuchalis*) provides an ideal system with which to study the function and evolution of avian vision. In this study we not only isolate and characterize the SWS1 pigment from *C. nuchalis* as a VS-type opsin, we also explore the function and evolution of recreated ancestral SWS1 pigments in passerines and parrots. We present experimental evidence indicating that although passerines and parrots evolved UVS by the same molecular mechanism, the passerine ancestor and parrot/passerine ancestor both had VS-type pigments, indicating UVS evolved independently in these two groups. We also investigate spectral tuning mutants of *C. nuchalis* SWS1, finding that λ_max_ is affected similarly by the mutations C86S, C86F and S90C as in other avian SWS1 opsins, suggesting spectral tuning in avian SWS1 pigments is unusually consistent compared to other vertebrate groups.

## Results

### Great bowerbird SWS1 spectral tuning mutants

**Figure 1:**
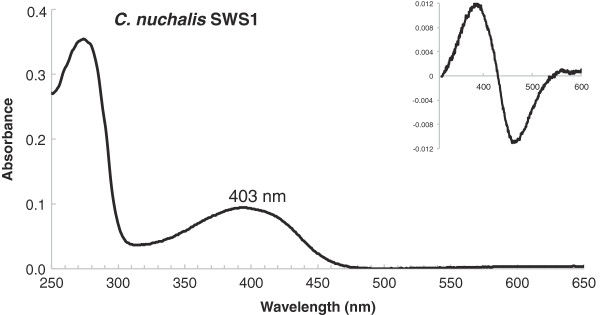
**UV-visible dark absorption spectrum of the wild type *C. nuchalis *SWS1.** Estimated absorption maximum values (λ_max_) noted above the dark spectrum. *Inset*, Dark-minus-acid bleached difference spectra.

**Figure 2:**
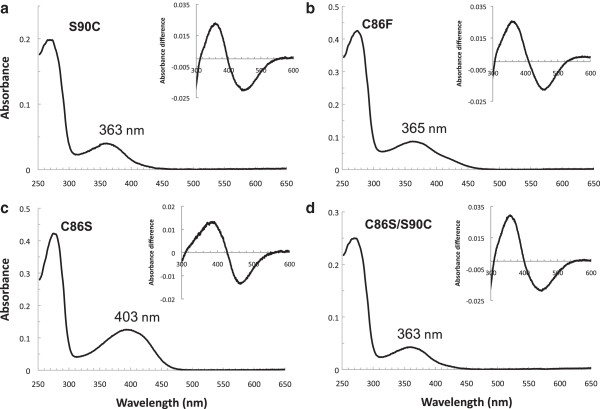
**UV-visible dark absorption spectra of *C. nuchalis *SWS1 mutants.** Dark spectra of **(A)** S90C, **(B)** C86F, **(C)** C86S, and **(D)** double mutant C86S/S90C, all recorded at pH 6.6. *Insets* show dark-minus-acid difference spectra. Estimated λ_max_ values indicated for each mutant.

**Figure 3:**
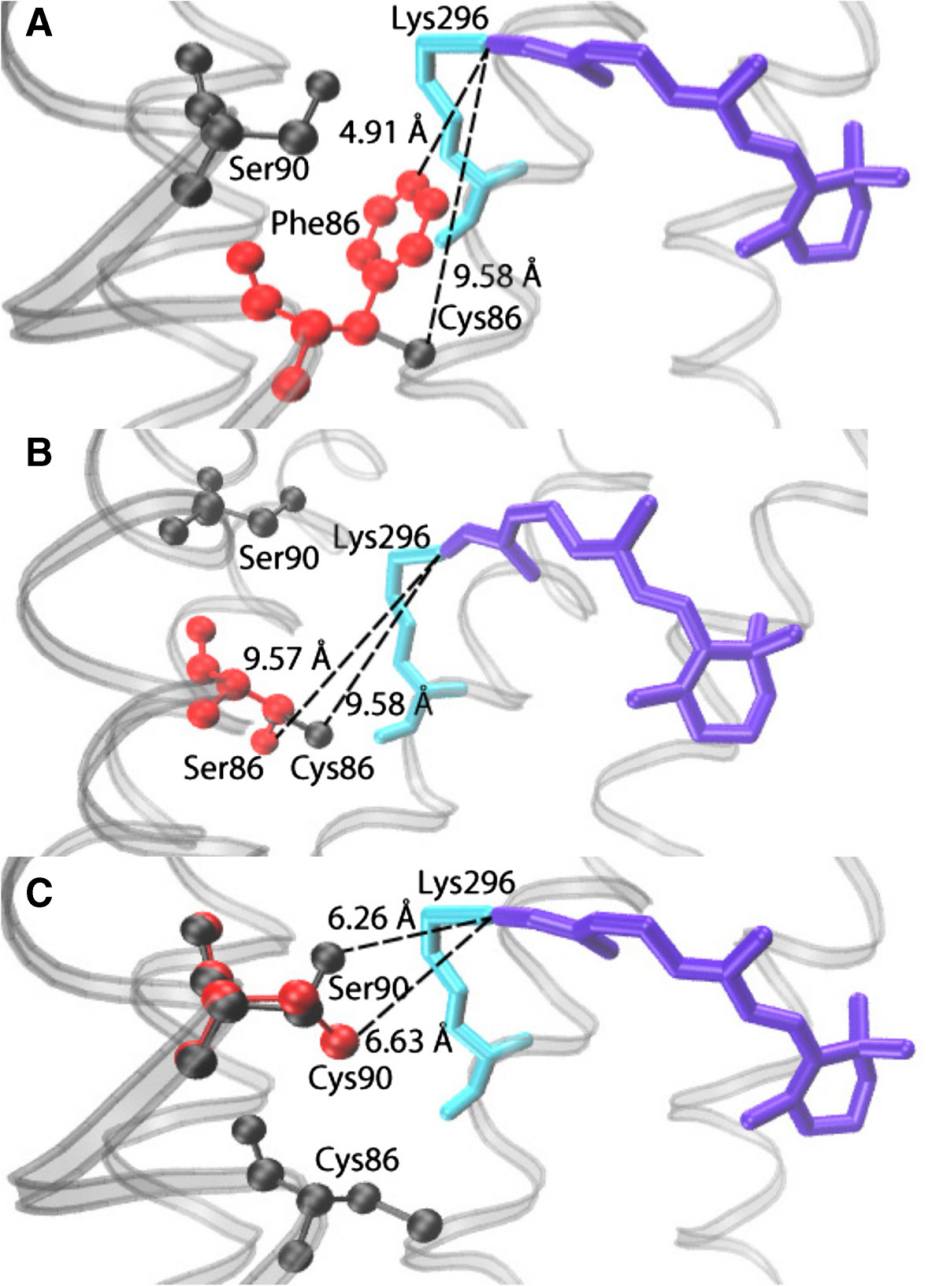
**Homology modeling of** ***C. nuchalis*** **SWS1.** Models are based on the bovine rhodopsin template, comparing the wild type structure with mutations **(A)** C86F, **(B)** C86S, and **(C)** S90C. Wild type residues are indicated in black, mutant residues in red. The 11-*cis* retinal chromphore is indicated in purple; with K296 in light blue, the site of chromophore attachment via a protonated Schiff base linkage. Estimated distances to the protonated Schiff base are indicated along the dotted lines.

The sequenced *C. nuchalis* SWS1 gene was found to contain amino acid residues C86 and S90, a combination found in past sequencing-surveys of avian SWS1 opsins [41,42], but one that has not been investigated in any *in vitro* expression and mutagenesis experiments. The expressed wild type bowerbird pigment was found to have a VS-type absorption spectrum (λ_max_ = 403 nm, Figure 1). This lies within the range of other expressed VS-type SWS1 avian opsins [17,18,28,43]. Mutating S90C in bowerbird SWS1 resulted in a UVS pigment (363 nm), with a 40 nm blue shift relative to wild type (Figure 2A). A similar effect was found with the C86F mutant, which also resulted in a UVS pigment (365 nm, Figure 2B). However, the mutation C86S had no effect (Figure 2C). The double mutant C86S/S90C had a λ_max_ at 363 nm, identical to the S90C single mutant (Figure 2D, Table 1). Homology modeling studies of bowerbird SWS1 structure confirm that there are only minor differences in side chain orientation for both C86S and S90C; however for C86F there is a large difference in side chain orientation, with F much closer to the protonated Schiff base (Figure 3).

**Table 1:** **Spectral absorbance characteristics for wild type *C. nuchalis *** **SWS1 pigments, site-directed mutants, and ancestral pigments**

**Pigment**	**λ**_**max**_^**a**^**(nm)**	**Shift from** ***C. nuchalis*** **wt pigment**^**b**^**(nm)**
GBS1 wt	402.97 ± 0.22	
S90C	363.05 ± 0.05	-40
C86S	403.05 ± 0.12	0
C86F^c^	365.72 ± 1.10	-37
C86S/S90C	362.95 ± 0.26	-40
Parrot/Passerine Ancestor	402.93 ± 0.28	0
Passerine Ancestor	404.28 ± 0.28	0

^a^ λmax values are given as mean ± standard deviation from at least three different measurements of dark absorbance spectra per expression. ^b^ λ_max_ shifts from *C. nuchalis* wild type (wt) pigment are expressed as negative for blue shifts. ^c^ λ_max_ of single mutant C86F calculated from fitting difference spectra of dark and acid denatured species.

**Figure 4:**
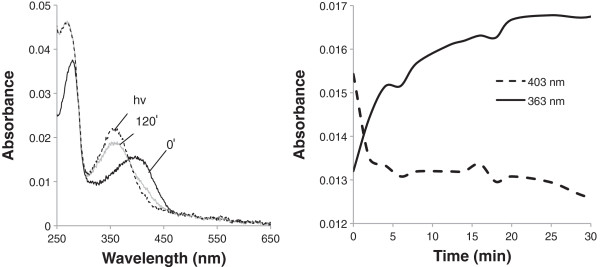
**Hydroxylamine reactivity of the *C. nuchalis *wild type SWS1 pigment.** Absorption spectra recorded t = 0 min after hydroxylamine addition (black line), and t = 120 min (grey line), followed by light bleaching (broken line). Right: The absorbance values at 403 nm (broken line) and 363 nm (black line) were plotted as a function of time after addition of hydroxylamine. Half-life for the formation of the retinal oxime in the presence of hydroxylamine was obtained by fitting the plot to a single exponential function.

Due to the short-wavelength λ_max_ of the bowerbird SWS1 and its mutants, a number of assays were performed in order to demonstrate a properly folded protein with bound 11-*cis* retinal chromophore, and to further characterize its function. In order to demonstrate a covalently bound chromophore, SWS1 pigments regenerated with 11-*cis* retinal were denatured in HCl, producing absorbance peaks shifted to 440 nm (Figures 1 &2, inset), characteristic of denatured opsin bound to chromophore [44]. All SWS1 pigments with λ_max_ above 400 nm were bleached with light to ~380 nm, characteristic of the biologically active state of visual pigments, metarhodopsin II [45,46]. Finally, the wild type bowerbird SWS1 pigment was found to react in the presence of hydroxylamine (Figure 4), with a t_1/2_ = ~6 min, typical of cone pigments [47,48].

Some of the pigments were occasionally found to have small secondary absorbance peaks in the longwave arm of the curve, which can have the effect of broadening pigment absorbance curves. These have previously been observed in wild type and mutant SWS1 pigments expressed in solution [17,18,20,24,49-51]. This has also been observed in blue shifted RH1 mutants with mutations at site 90 [51-53]. In this study, experimental attempts to narrow the absorption spectra, including the use of TRIS phosphate buffers, exclusion of glycerol, decreasing purification time and minimizing light and temperature exposure, were unsuccessful, similar to previous experimental studies [18,50,51,54].

In addition to SWS1, four other opsin genes were also isolated from *C. nuchalis*: SWS2, RH2, and LWS, and rod opsin (RH1) (Additional file 1: Figure S2). All opsin genes were found to contain important structural characteristics typical of functional visual pigments. Phylogenetic analyses show these sequences cluster with expected visual pigment families (Additional file 1: Figure S3).

### Reconstructing passerine and parrot/passerine ancestral SWS1 pigments

**Figure 5:**
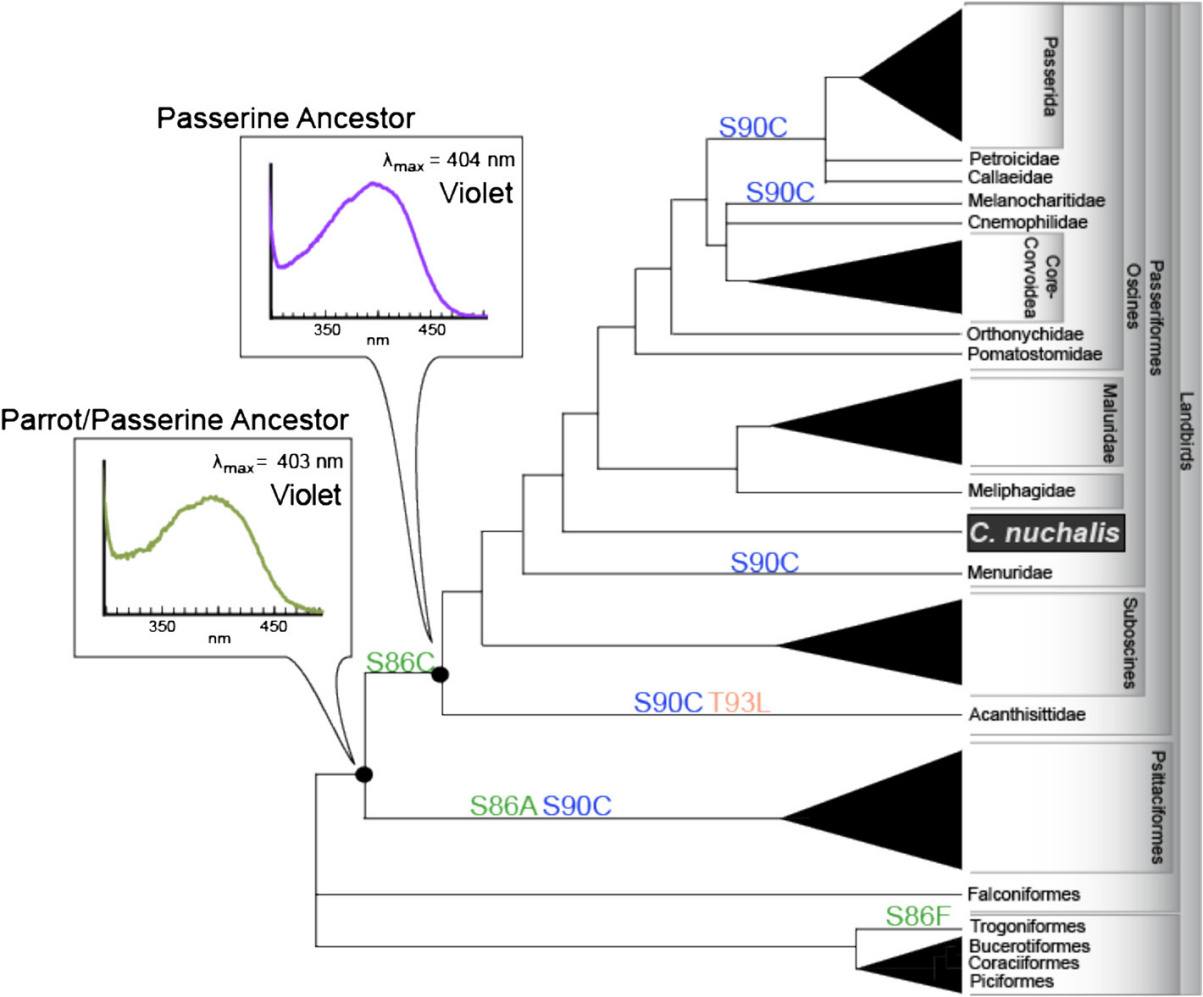
**SWS1 visual pigment evolution, with ancestrally reconstructed substitutions at sites 86, 90 & 93 mapped on a phylogeny of Landbirds **[31-33,40,56,80-87]**.** Experimentally reconstructed ancestral nodes are shown along with measured λ_max_ values. GenBank accession numbers provided in Additional file 1: Table S2.

In order to investigate the evolution of UV sensitivity in passerines and parrots, a combination of Bayesian and maximum likelihood ancestral reconstruction methods were used to infer the sequence of Helix 2 of SWS1 in the ancestors of passerines and parrots (Additional file 1: Table S4). Reconstructed amino acid substitutions at major spectral tuning sites were mapped on a landbird phylogeny (Figure 5). Relative to site 90, less variation was found at sites 86 and 93, with a notable substitution, S86C, occurring at the base of the passerine lineage. Interestingly, substitutions at site 90 were found to occur multiple times throughout the passerine phylogeny, and always involve a change from S to C, suggestive of multiple shifts towards UV sensitivity (but not the reverse). This finding is in disagreement with a previous study proposing that the residue at site 90 has transitioned back and forth between S and C multiple times throughout passerine evolution [41]. Their results would suggest that transitions between UV and violet pigments are quite labile, whereas our results would imply more constrained evolution.

**Figure 6:**
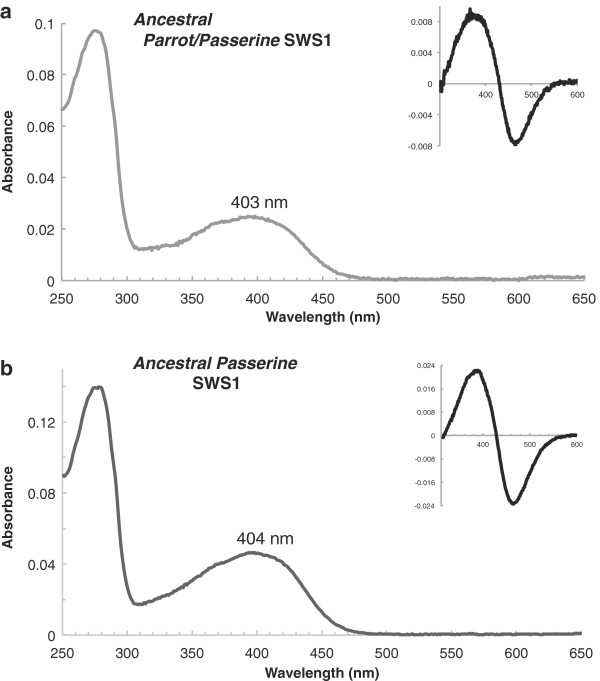
**UV-visible dark absorption spectra of the (A) ancestral SWS1 pigment of passerines and parrots and (B) ancestral SWS1 pigment of passerines.** Absorption maxima (λ_max_) noted above the dark spectra. *Inset*, dark-minus-acid bleached difference spectra.

SWS1 pigments for the ancestors of the passerines and parrots were experimentally recreated in the background of our *C. nuchalis* pigment. This was done for a number of reasons. First, we were limited by current sequence data, which only exists for Helix 2 for most bird SWS1 genes, as all known spectral tuning sites are thought to be contained in this helix. Second, as a basal passerine, *C. nuchalis* SWS1 differed from the reconstructed ancestral sequences at specific sites in Helix 2, allowing us to generate the ancestrally reconstructed sequences using site-directed mutagenesis methods. Third, our ability to make direct functional comparisons between the ancestral pigments and that of *C. nuchalis* allowed us to better interpret the effects of particular amino acid substitutions. The experimentally assayed, recreated ancestral SWS1 pigments were both found to be VS pigments, absorbing maximally in the VS at 403 nm (parrot/passerine ancestor) and 404 nm (passerine ancestor, Figure 6). Both ancestral pigments were found not only to bind retinal, but also to activate in response to light and denature in acid (Figure 6, inset). The reconstructed nodes had high posterior probability values across sites (Additional file 1: Table S4). Reconstructions on an alternate topology favored by previous visual pigment studies [41] did not find any differences with our experimentally recreated sequences (Additional file 1: Figure S4).

## Discussion

This study extends our understanding of SWS1 opsin function and evolution by investigating evolutionary changes that occurred in avian SWS1 genes. The SWS1 opsin of the great bowerbird *C. nuchalis*, a basal passerine bird, was expressed along with a series of spectral tuning mutants and ancestral passerine SWS1 pigments allowing us to investigate spectral tuning mechanisms and identify the evolution of UV/violet sensitivity in early passerines and parrots. The *C. nuchalis* SWS1 opsin was found to be a VS pigment, with a maximal absorbance of 403 nm, which is in agreement with previous MSP studies identifying a λ_max_ of 404 nm [39]. However, our experimentally recreated passerine ancestral SWS1 pigments were also found to be VS, addressing a longstanding issue of ancestral passerine SWS1 spectral tuning in previous studies [25,28,41,55].

### Evolution of UV/violet vision in passerines and parrots

Our finding that the passerine ancestor had a violet-type SWS1 reaches slightly different conclusions in comparison with a recent study suggesting that the passerine ancestor was UVS [41], which was the first paper examining avian SWS1 evolution that used a phylogeny in which passerines and parrots were specified sister orders. Not only are the predicted ancestral sequences different, but a VS-type λ_max_ in ancestral pigment was experimentally confirmed in our study. While it is not entirely clear why our study reached such different conclusions, there are a number of important differences. Our analysis included additional outgroup sequences, and used maximum likelihood reconstruction methods (as opposed to parsimony). Furthermore, in our study the ancestral pigments were experimentally recreated and functionally assayed. Finally, our phylogeny is based on the current understanding of phylogenetic relationships among landbirds that includes a recent revision of the relationships among higher lineages [56-58], and therefore is somewhat different from that of Odeen *et al*. [41]. However, we did not find any differences in our reconstructions of the ancestral passerine SWS1 when we used a tree with the relationships among higher passerines arranged similar to their phylogeny, suggesting that the difference in our findings from previous studies are probably due to methodological differences, such as the use of maximum likelihood reconstruction methods and/or the use of additional outgroup lineages. (Odeen *et al*. [41] did note that the inclusion of additional outgroup sequences resulted in an ambiguous reconstruction of the passerine ancestor even in their analyses.) Our results support earlier studies that investigated the evolution of UV/violet sensitivity in birds suggesting the passerine ancestor had a VS type SWS1 [25,28,55], but these early studies do not place passerines and parrots as sister orders. Because the parrots are now thought to be closer to the basal passerines than before, our results are more robust than they would be if based upon the older tree.

Our findings, that UVS in passerines and parrots evolved from VS ancestors, and that this occurred independently in at least two lineages, are rather unusual with respect to other vertebrate groups. The ancestral vertebrate state is thought to have been UVS, with VS pigments evolving independently in various lineages within fish, mammals, and amphibians [16,22-24,28,29,50]. Birds are believed to be an interesting exception where a switch to VS is thought to have occurred in the ancestral avian pigment with some descendants subsequently re-evolving UVS [24,50]. Our identification of VS type pigments in both passerine and parrot/passerine ancestors confirm this hypothesis, and our ancestral reconstruction results provide a more precise prediction of where these spectral sensitivity shifts occurred. The re-evolution of UVS from a VS type pigment has not previously been predicted elsewhere in the vertebrate phylogeny. The reasons why bird SWS1 pigments are an exception remain largely unknown, but may be related to their unique spectral tuning mechanisms among vertebrates.

### Spectral tuning in C. nuchalis SWS1

The *C. nuchalis* VS pigment possesses an unusual residue combination at the two spectral tuning sites known to be most important in specifying UVS or VS in vertebrates: C86/S90. This residue combination has been found in a few passerine SWS1 opsins in past sequence-based surveys [41,42], but its spectral relevance has not been examined using mutagenesis experiments, which thus far have only dealt with VS-type pigments with S86/S90, in pigeon and chicken, [18,28] and UVS type with either A86/C90 or C86/C90, in budgerigar and zebra finch, respectively [17,18,59]. Past mutagenesis studies of vertebrate SWS1 pigments have shown the magnitude of λ_max_ shift caused by a given amino acid change can differ significantly among pigments due to synergistic interactions within and between transmembrane regions I-VII [19,50,60,61]. Characterization of *C. nuchalis* SWS1 mutants was therefore carried out, as it may provide new clarification of the mechanisms contributing to the naturally occurring variation in avian SWS1 pigment spectral sensitivity, particularly among the VS type pigments. These mutants can also help clarify patterns of evolution between VS and UVS visual systems in birds.

Our results showing that S90C shifts the *C. nuchalis* SWS1 into the UV is consistent with previous studies where similar shifts have been documented in the chicken, pigeon, and the reverse in zebra finch, and budgerigar [17,18,28]. In *C. nuchalis*, the effect of the double mutant C86S/S90C was identical to that of the single S90C mutant. Thus, in the presence of C90, C86 has no additional effect on sensitivity. In other avian pigments, substitutions at known spectral tuning sites also do not change λ_max_ if expressed with C90 [17,28]. Others have suggested that the effect of C90 is so strong it prevents detection of any subtler effects other residues might have [28]. In birds, all *in vitro* expressed pigments, whether wild type or mutant, with C90 have λ_max_ ~360 nm. The exception is in chicken where S90C only shifts λ_max_ to 369 nm [18].

The mutation C86F in *C. nuchalis* also shifts λ_max_ into the UV. Unlike C90, which, as far as we know only has a functional role in avian SWS1 opsins, F86 is an important spectral tuning site across vertebrates where it confers UVS in most pigments in which it occurs [16,19,29,30], the exception being the aye-aye, which is VS despite the presence of F86 [23]. It is, in fact, believed to be the ancestral vertebrate state and substitutions from F86 are responsible for the loss of UVS in many mammalian lineages [16,19,22,23,29,30], and in ancient birds [21]. In *C. nuchalis*, C86 therefore plays an important role in maintaining sensitivity in the violet range, as the replacement of C86F shifts λ_max_ into the ancestral UV state. F86 is also interesting because it has been suggested to be a second mechanism by which birds achieve UVS: It is found in the SWS1 genes of some birds including the trogon, paleognaths and a few sandgrouses and motmots [25-27], is capable of UV shifting VS pigments of pigeon and chicken [28], and is responsible for UVS in fish and most mammals [19,29,62]. Correspondingly, our mutagenesis results support the hypothesis that extant birds with F86 are UVS, and, therefore, the supposition that there are at least two mechanisms determining UVS in birds [28]. The expression of a wild type pigment with F86 would be needed to confirm this hypothesis.

In contrast to the previous mutants, C86S did not affect λ_max_ in the *C. nuchalis* SWS1. This mutation was previously suggested as contributing to the broad spectral variation observed among VS type pigments [55,59], which in birds range from 388 nm (pigeon) to 420 nm (chicken) [28]. Site 86 is an important spectral tuning site in other vertebrate SWS1 pigments, and S86C is capable of shifting λ_max_ into the UV in a hypothetical ancestral avian SWS1 [21]. As with *C. nuchalis* SWS1, S86C barely shifts λ_max_ in the pigeon SWS1 [28], and mutation to serine at site 86 has no effect on the budgerigar SWS1 [17]. Therefore the residues responsible for this large variation in λ_max_ among VS pigments remain unknown. Altogether, these studies indicate that the role of site 86 in avian SWS1 pigments depends not only on the residue at that site, but also on the background in which it is expressed. This is particularly true of mammalian SWS1 pigments where the variation at site 86 is better characterized: in most mammalian pigments the presence of F86 dramatically shifts λ_max_, into the UV [16,19,29,30], but this is not always the case [23].

### Implications for behavioural ecology

While higher passerine lineages with UV type pigments are known to use UV signals in communication [9-11], current evidence indicates no link between colouration and spectral sensitivity in bowerbirds [39]. Here we have shown that despite the fact males display UV reflecting feathers and objects during courtship [3,63,64], *C. nuchalis* does not possess a UV type SWS1 visual pigment. These findings would seem to contradict evidence demonstrating a strong link between spectral tuning and signal colouration in other vertebrate groups, [65,66], and the belief that UV type pigments offer a dramatic advantage by improving sensitivity in this short wave range [67].

The general correlation between colouration and sensitivity remains because birds with VS pigments can perceive UV; SWS1 visual pigments absorb strongly over most of the UV visible range [6], cone oil droplets are effectively transparent to light in this range [68] and, in most species, avian ocular media transmit most short wavelength light [69]. The difference in UV sensitivity between UVS, VS and the blue shifted bowerbird VS is just a matter of degree. Nevertheless, while UV colouration might be perceived by bowerbirds, its importance in communication is not well understood. In the satin bowerbird (*Ptilonorhynchus violaceus*) plumage UV reflectance is correlated with factors such as the intensity of infection from blood parasites, feather growth rate, and body size [63], but it is unrelated to mating success [64].

Given that *C. nuchalis* and other bowerbird ocular media transmit more UV wavelengths than most other species with VS-type visual pigments, they might represent a transitional link in the evolution from a VS to a UVS visual system [39]. This hypothesis is supported by the comparatively blue shifted SWS1 found in bowerbirds, which further augments UV sensitivity. Given the similarly blue shifted λ_max_ of the ancestral SWS1 pigments, this hypothesized transitional state might have originated in the ancestral passerine, and be shared among other basal passerines as well. This could also explain the unusually high number of shifts from VS to UVS in this order. Further investigation into the evolutionary history of ocular transmission would be useful to clarify this possibility.

If an organism with a blue shifted VS pigment, like the great bowerbird, has sufficient UV sensitivity, then the adaptive advantage of a switch to UVS might not be as large as it would be if it could perceive little UV or only had the ancestral VS pigment. Aside from λ_max_, there are a number of other structural and functional differences between VS and UVS opsins that may be related to a deprotonated Schiff base linkage to the chromophore [48,51,70-74]. These differences may have important consequences for the evolution of UVS in birds and other vertebrates. Therefore, it is possible that the wavelength difference between UVS and VS type pigments might not be the only, or the most important, functional difference between them. Further biochemical and mutagenesis studies would be necessary to refine the functional differences between these two opsin subtypes.

## Conclusions

Our *in vitro* experiments suggest that spectral tuning in *C. nuchalis* is likely mediated by mechanisms very similar to those of other birds. This is unusual relative to spectral tuning mechanisms within mammals, which vary considerably among and within the major mammalian orders. In addition, despite both parrots and passerines sharing UV sensitivity and the same spectral tuning mechanism the experimentally recreated ancestral passerine and parrot/passerine SWS1 pigments were both found to be maximally sensitive in the violet; this suggests that UV sensitivity may have evolved independently in passerines and parrots from a violet sensitive ancestor. Moreover, our ancestral sequence reconstructions of SWS1 in landbird evolution suggest that transitions from VS to UVS are much more likely than the reverse. Our ancestral reconstruction experiments allow for a more precise prediction of where spectral sensitivity shifts may have occurred, and provide an unusual example where descendants have re-evolved UVS from a violet type ancestor; the reverse being more common in most vertebrates.

## Methods

### Opsin sequences

Birds were collected using cage traps or mist nets under appropriate Australian (Queensland Parks and Wildlife F1/000331/00/SAA, Australian Bird and Bat Banding Scheme 2434,1310, Commonwealth Scientific, Industrial and Research Organization (CSIRO) Ethics OB15/12, James Cook University Ethics A562, United States Department of Agriculture 47746, Australian Quarantine and Inspection Station 200104468, Environment Australia PWS P20011711, Department of Natural Resources Australia 1576) and US permits and authorizations (UCSB IACUC #10-98-555-1, USDA 47746). Birds were euthanized according to these protocols. Retinas were preserved in RNA Later (Invitrogen), and stored on ice in the field until they could be transferred to -80 for long term storage. RNA was extracted from retinal tissue using TRIzol Reagent (Invitrogen), and a cDNA library was prepared with the SMART cDNA Library Construction Kit (BD Biosciences). Degenerate primers were designed to amplify fragments of the opsin coding regions (Additional file 1: Table S1), with 3′ and 5′ ends of the genes isolated by RACE PCR. Purified PCR products were cloned into pJET1.2 (Fermentas), and sequenced from multiple clones. Site-directed mutagenesis was performed using the QuikChange kit (Stratagene). Blood samples of two individuals (“T + EB” & “BG/Z”) found in the Lavarack Barracks military base in Townsville City Queensland, Australia were preserved in Queen’s lysis buffer (0.01 M Tris, 0.01 M NaCl, 0.01 M sodium EDTA, and 1.0% *n*-lauroylsarcosine, pH 8.0) [75]. Genomic DNA was extracted from these blood samples using the DNeasy Blood and Tissue Kit (Qiagen). Introns and flanking genomic regions were isolated using PCR with specific primers on a genomic library created with the Genome Walker kit (Clontech).

### Expression & purification of wild type and mutant pigments

Full-length coding sequences of *C. nuchalis* wild type pigments were amplified from cDNA, and cloned into the p1D4-hrGFP II expression vector for transient expression [76]. This vector has a C-terminal 1D4 epitope tag that encodes the last nine amino acids of bovine RH1 [TETSQVAPA], and employs the CMV promoter to drive transgene expression. Cultured HEK293T cells were transiently transfected with the opsin-1D4 construct using the Lipofectamine 2000 reagent (Invitrogen). Typically four 175 cm^2^ flasks were used per SWS1 expression procedure, with one flask of similarly expressed bovine rhodopsin as a control. Methods for purification of *C. nuchalis* SWS1 opsins were adapted from those of Starace & Knox [77]. Briefly, cells were harvested, washed with Harvesting Buffer (50 mM HEPES ph 6.6, 140 mM NaCl, 3 mM MgCl_2_), regenerated with 11-*cis* retinal chromophore, solubilized (in 1% *n*-dodecyl-β-D-maltopyranoside detergent (DM) with 20% (w/v) glycerol), and purified by batch immunoaffinity chromatography with the 1D4 monoclonal antibody [78]. The UV-visible absorption spectra of purified visual pigments were recorded at 21°C using a Cary 4000 dual beam spectrophotometer (Agilent). For functional assays, absorbance spectra were also measured after exposure to light (either a 366 nm UV light illuminator for UVS pigments, or a 60-W lamp with 440 nm cutoff filter for VS pigments), to hydrochloric acid (HCl; 100 mM), or to hydroxylamine (NH_2_OH; 50 mM). To produce difference spectra, either the light or the acid-denatured spectra were subtracted from the dark absorbance spectra. To estimate λ_max_, the dark absorbance spectra were baseline corrected and fit to a visual pigment template [73]. The F86 mutant λ_max_ was estimated by fitting the dark-acid difference spectrum [29], due to a perturbation in the long wave arm of the dark spectrum. All amino acid numbering in this manuscript is according to the bovine rhodopsin amino acid sequence as a reference.

### Ancestral sequence reconstruction

To reconstruct ancestral passerine SWS1 sequences, a dataset of 83 SWS1 genes from passerines, parrots and other related landbirds, as per Hackett *et al*. [31], was assembled from GenBank for a region of Helix 2 that encompasses all the known SWS1 spectral tuning sites (Additional file 1: Table S2 and Figure S1). For the majority of sequences, this region is the only portion of the SWS1 gene for which sequence data is available. The sequences were aligned with our *C. nuchalis* sequence using PRANK ([79], Figure S1). For ancestral reconstruction, a topology reflecting current understanding of landbird relationships was used (Figure 5) [31-33,40,56,80-87]. This phylogeny incorporates recent information that places passerines and parrots as derived sister orders relative to other Landbird orders [31-33], and includes a recent revision of the relationships among higher passerine lineages [56-58]. This phylogeny is somewhat different from previous avian SWS1 studies, therefore we also analyzed our data on an alternate phylogeny (Additional file 1: Figure S4) similar to that of Odeen *et al*. [41], in order to investigate the robustness of our ancestral reconstructions.

For the ancestral sequence reconstruction (ASR), a combination of empirical Bayesian and maximum likelihood (ML) codon-based methods [88] were used (PAML v4.3 [89]). Nested random sites codon models were compared using likelihood ratio tests (LRTs) [90,91], and the best fitting model, M7 [92], was used for the ancestral sequence reconstruction (Additional file 1: Table S3 and S4). Multiple runs were carried out with different starting values to check for convergence in all analyses. In experimentally resurrecting ancestral proteins, focusing solely on the most probable ancestral sequence can introduce biases in amino acid composition, which may in turn alter the functional phenotype of a resurrected protein [93,94]. We addressed this concern using a strategy of weighted random sampling of ancestral sequences from the posterior distribution, in order to avoid this bias [94,95]. For the two ancestral nodes reconstructed, a weighted sampling of 10,000 sequences from the posterior distribution resulted in ancestral sequences that were either identical (parrot/passerine ancestor, 100% identical), or highly similar to (passerine ancestor, 83% or 8343 sequences out of 10,000 identical) the most likely ancestral reconstruction.

### Homology modeling

The 3D structure of the *C. nuchalis* wild-type SWS1 was inferred via homology modeling by Modeller [96], using bovine rhodopsin (PDB code: 1U19, [97]) as template. Fifty models were generated by optimizing the Modeller objective function with the model with the lowest DOPE score [98] selected for further assessment and visualization. Model quality was checked using ProSA-web [99] to ensure the model and template structures have comparable z-scores (an standardized indicator of a structure’s total energy compared to that expected by random chance), and by ProCheck [100], to ensure bond lengths and angles do not have unusual stereochemical conformations. Similar procedures were followed for inferring 3D structures of C86F, C86S and S90C mutants.

## Abbreviations

SWS1: Short-wavelength sensitive 1; UV: Ultraviolet; V: Violet; λmax: Wavelength of maximum absorbance.

## Competing interests

The authors declare that they have no competing interests.

## Authors’ contributions

BSWC, JAE and IvH conceived of the study. IvH performed the lab work, compiled the data, performed analyses and drafted the manuscript. AS performed the homology modeling and structural analyses, and LD collected the samples. BSWC guided all aspects of the study, and helped to draft the manuscript. All authors contributed to the final version of the manuscript.

## Supplementary Material

Additional file 1: Table S1 Degenerate oligonucleotides for PCR (numbering according to bovine rhodopsin). **Table S2**. Species names & accession numbers for Landbird SWS1 data set used in ancestral reconstruction analysis. **Table S3**. Likelihood scores of codon models used for ancestral reconstruction. **Table S4**. Maximum likelihood ancestral reconstruction of ancestral passerine/parrot, and ancestral passerine SWS1 pigments, with posterior probabilities (numbering according to bovine rhodo). **Figure S1**. Alignment of SWS1 opsin gene, helix 2 from Landbirds used in ancestral reconstruction, highlighting sites 86, 90 & 93. **Figure S2**. Alignment of visual pigment sequences in *C. nuchalis*. **Figure S3**. Phylogenetic relationships of the *C. nuchalis* opsin genes with those of other vertebrates. **Figure S4**. Alternate Landbird topologies used to confirm ancestral sequence reconstruction [101-108]. Click here for file
