# Supplementary material for: Functional characterization of spectral tuning mechanisms in the great bowerbird short-wavelength sensitive visual pigment (SWS1), and the origins of UV/violet vision in passerines and parrots
Source: BMC Evol Biol. 2013 Nov 13;13:250. doi: 10.1186/1471-2148-13-250 (PMC4029201; doi:10.1186/1471-2148-13-250)
Supplement: Additional file 1: Table S1 — Degenerate oligonucleotides for PCR (numbering according to bovine rhodopsin). Table S2. Species names & accession numbers for Landbird SWS1 data set used in ancestral reconstruction analysis. Table S3. Likelihood scores of codon models used for ancestral reconstruction. Table S4. Maximum likelihood ancestral reconstruction of ancestral passerine/parrot, and ancestral passerine SWS1 pigments, with posterior probabilities (numbering according to bovine rhodo). Figure S1. Alignment of SWS1 opsin gene, helix 2 from Landbirds used in ancestral reconstruction, highlighting sites 86, 90 & 93. Figure S2. Alignment of visual pigment sequences in C. nuchalis. Figure S3. Phylogenetic relationships of the C. nuchalis opsin genes with those of other vertebrates. Figure S4. Alternate Landbird topologies used to confirm ancestral sequence reconstruction [101-108]. [file 1471-2148-13-250-S1.pdf]

Supplementary data

Supplementary tables

Table S1. Degenerate oligonucleotides for PCR (numbering according to bovine rhodopsin)

Name	Target opsin	Sequence 5' - 3'	Direction of primer	AA site
AvesRH2_1018R	RH2	gac gga sga gac ctc rgt ytt gct	R	1018
AvesRH2_898R	RH2	tca tga gsa crt aga tga ygg ggt t	R	898
AvesRH2_179F	RH2	tgg tca cct tca arc aca aga agc t	F	179
AvesRH2_785R	RH2	gcr tag ggy gtc cag gcc agc at	R	785
VertS2_103R	SWS2	aag tac ats tgg gag aag ctg ta	R	103
VertS2_315R	SWS2	cga vcg gaa ctg ytk gtt cat	R	315
VertS2_95F	SWS2	ttc tac agc ttc tcs cag atg tac tt	F	95
VertS2_22F	SWS2	agc ccn ttc ctg gtv ccs ca	F	22
AvesS2_258R	SWS2	cac cat cac vay yac cat cyt sgt	R	258
VertUV_243F	SWS1	acg cag aag gci gag mri gar gt	F	243
VertUV_232F	SWS1	gcc gtg gcc gci car car car ga	F	232
TetUV_187F	SWS1	tgc gcc cig act cct aya cn	F	187
VertUV_230R	SWS1	ctg ctg cgc cgc iac igc nyk nag	R	237
TetUV_239R	SWS1	tcc tgc tgc tgi gci gcn acn gc	R	239
AvesUV_306R	SWS1	gaa ctg ctt gtt cat raa rca rta	R	313
VertUV_230F	SWS1	ctg cgc gcc gtc gci gcn car ca	F	230
AvesUV_306R	SWS1	gaa ctg ctt gtt cat raa rca rta	R	306

Table S2. Species names & accession numbers for Landbird SWS1 data set used in ancestral reconstruction analysis:

Order	Family	Species	Common Name	Accession #	Reference	Name used in phylogeny
Bucerotiformes	Upupidae	*Upupa epops*	Eurasian hoopoe	AY227191	(Ödeen and Håstad 2003)	Upupa
Coraciiformes	Alcedinidae	*Alcedo atthis*	common kingfisher	AY227169	(Ödeen and Håstad 2003)	Alcedo
Coraciiformes	Coraciidae	*Coracias garrulus*	common roller	AY227170	(Ödeen and Håstad 2003)	Coracias
Piciformes	Picidae	*Dendrocopos major*	great spotted woodpecker	AY227184	(Ödeen and Håstad 2003)	Dendrocop
Trogoniformes	Trogonidae	*Trogon curucui*	blue-crowned trogon	AY227190	(Ödeen and Håstad 2003)	Trogon
Falconiformes	Falconidae	*Falco peregrinus*	peregrine falcon	AY227157	(Ödeen and Håstad 2003)	Falco
Passeriformes	Corvidae	*Corvus corone cornix*	hooded crow	AY227176	(Ödeen and Håstad 2003)	CorvusC
Passeriformes	Corvidae	*Pica pica*	Eurasian magpie	GQ305970	(Ödeen and Håstad 2009)	Pica
Passeriformes	Vireonidae	*Cyclarhis gujanensis*	rufous-browed peppershrike	HE601826	(Ödeen, et al. 2011)	Cyclarhis
Passeriformes	Oriolidae	*Oriolus oriolus*	Eurasian golden oriole	HE601828	(Ödeen, et al. 2011)	Oriolus
Passeriformes	Paradisaeidae	*Ptiloris magnificus*	magnificent riflebird	HE601835	(Ödeen, et al. 2011)	Ptiloris
Passeriformes	Corvidae	*Corvus frugilegus*	rook	DQ451006	Browne et al. (Genbank 2006)	CorvusF
Passeriformes	Campephagidae	*Coracina novaehollandiae*	black-faced cuckooshrike	HE601825	(Odeen, et al. 2012)	Coracina
Passeriformes	Dicruridae	*Dicrurus bracteatus*	spangled drongo	HE601829	(Odeen, et al. 2012)	Dicrurus
Passeriformes	Corvidae	*Cyanocorax chrysops*	plush-crested jay	HE601832	(Odeen, et al. 2012)	Cyanocora
Passeriformes	Dicruridae	*Rhipidura fuliginosa*	New Zealand fantail	HM159132	Hauber & Chong (GenBank 2010)	Rhipidura
Passeriformes	Callaetidae	*Creadion carunculatus*	saddleback	HM159129	Hauber & Chong (GenBank 2010)	Creadion
Passeriformes	Meliphagidae	*Lichenostomus flavescens*	yellow-tinted honeyeater	GQ305955	(Ödeen and Håstad 2009)	Lichenost
Passeriformes	Maluridae	*Acanthorhynchus tenuirostris*	eastern spinebill	GQ305959	(Ödeen and Håstad 2009)	Acanthorh
Passeriformes	Maluridae	*Malurus grayi*	broad-billed fairywren	HE588093	(Odeen, et al. 2012)	MalurusG
Passeriformes	Maluridae	*Malurus cyanocephalus*	emperor fairywren	HE588094	(Odeen, et al. 2012)	MalarusCy
Passeriformes	Maluridae	*Malurus amabilis*	loverly fairywren	HE588095	(Odeen, et al. 2012)	MalarusA
Passeriformes	Maluridae	*Malurus elegans*	red-winged fairywren	HE588099	(Odeen, et al. 2012)	MalurusE
Passeriformes	Maluridae	*Malurus splendendens musgravi*	turquoise fairywren	HE588101	(Odeen, et al. 2012)	MalurusS
Passeriformes	Maluridae	*Malurus coronatus*	purple-crowned fairywren	HE588108, HE588109	(Odeen, et al. 2012)	MalurusCo
Passeriformes	Maluridae	*Malurus melanocephalus*	red-backed fairywren	HE588111-HE588113	(Odeen, et al. 2012)	MalurusM
Passeriformes	Maluridae	*Malurus leucopterus edouradi*	white-winged fairywren	HE588114-HE588116	(Odeen, et al. 2012)	MalurusL
Passeriformes	Maluridae	*Clytomias insignis*	orange crowned fairywren	HE588121	(Odeen, et al. 2012)	Clytomyia
Passeriformes	Maluridae	*Amytorins barbatus*	grey grasswren	HE588123	(Odeen, et al. 2012)	Amytornis
Passeriformes	Acanthizidae	*Gerygone igata*	grey gerygone	HM159130,	Hauber & Chong (GenBank	Gerygone

				HM159131	2010)	
Passeriformes	Menuridae	*Menura novaehollandiae*	superb lyrebird	HE601819	(Ödeen, et al. 2011)	Menura
Passeriformes	Ptilonorhynchidae	*Sericulus chrysocephalus*	regent catbird	HE588091	(Ödeen, et al. 2011)	Sericulus
Passeriformes	Pomatostomidae	*Pomatostomus temporalis*	grey-crowned babbler	HE601820	(Ödeen, et al. 2011)	PomatostT
Passeriformes	Pomatostomidae	*Pomatostomus ruficeps*	chestnut-crowned babbler	HE601821	(Ödeen, et al. 2011)	PomatostR
Passeriformes	Orthonychidae	*Orthonyx temminckii*	Australian logrunner	HE601822	(Ödeen, et al. 2011)	Orthonyx
Passeriformes	Cnemophilidae	*Cnemophilus loriae*	Loria's satinbird	HE601823	(Ödeen, et al. 2011)	Cnemophil
Passeriformes	Melanocharitidae	*Toxorhamphus poliopterus*	slaty-headed longbill	HE601824	(Ödeen, et al. 2011)	Toxorham
Passeriformes	Ptilonorhynchidae	*Chlamydera nuchalis*	great bowerbird		This Study	Chlamyder
Passeriformes	Sittidae	*Sitta europaea*	Eurasian nuthatch	HE601851, HE601852	(Ödeen, et al. 2011)	Sitta
Passeriformes	Sturnidae	*Sturnus vulgaris*	common starling	AY227180	(Ödeen and Håstad 2003)	Sturnus
Passeriformes	Muscicapidae	*Luscinia svecica*	bluethroat	AY274225	Raman & Andersson (Genbank 2003)	LusciniaS
Passeriformes	Muscicapidae	*Luscinia calliope*	Siberian rubythroat	AY274226	Raman & Andersson (Genbank 2003)	LusciniaC
Passeriformes	Mimidae	*Mimus saturninus*	chalk-browed mockingbird	GQ305972	(Ödeen and Håstad 2009)	Mimus
Passeriformes	Turdidae	*Turdus iliacus*	redwing	HE601854	(Ödeen, et al. 2011)	Turdus
Passeriformes	Paridae	*Parus caeruleus*	blue tit	AY274220	Raman & Andersson (Genbank 2003)	
Passeriformes	Paridae	*Parus major*	great tit	AY274221	Raman & Andersson (Genbank 2003)	Parus
Passeriformes	Paridae	*Parus palustris*	marsh tit	AY274222	Raman & Andersson (Genbank 2003)	
Passeriformes	Passeridae	*Taeniopygia guttata*	zebra finch	AF222331	(Yokoyama, et al. 2000)	Taeniopyg
Passeriformes	Fringillidae	*Serinus canaria*	common canary	AJ277922	(Das, et al. 1999)	Serinus
Passeriformes	Passeridae	*Euplectes afer*	yellow crowned bishop	AY274223	Raman & Andersson (Genbank 2003)	Euplectes
Passeriformes	Estrilidae	*Amadina fasciata*	cut-throat finch	FJ440639	(Ödeen and Håstad 2009)	Amadina
Passeriformes	Estrilidae	*Lonchura maja*	white-headed munia	FJ440641	(Ödeen and Håstad 2009)	Lonchura
Passeriformes	Passeridae	*Neochmia modesta*	plum-headed finch	FJ440642	(Ödeen and Håstad 2009)	Neichima
Passeriformes	Nectariniidae	*Cinnyris pulchellus*	beautiful sunbird	GQ305964	(Ödeen and Håstad 2009)	Cinnyris
Passeriformes	Motacillidae	*Anthus cervinus*	red-throated pipit	HE601865	(Ödeen, et al. 2011)	Anthus
Passeriformes	Icteridae	*Sturnella neglecta*	Western meadowlark	HE601868	(Ödeen, et al. 2011)	Sturnella
Passeriformes	Phylloscopidae	*Phylloscopus trochilus*	willow warbler	AY227181	(Ödeen and Håstad 2003)	Phyllosco
Passeriformes	Timaliidae	*Leiothrix lutea*	red-billed leiothrix	FJ440645	(Ödeen and Håstad 2009)	Leiothrix
Passeriformes	Hirundinidae	*Hirundo rustica*	barn swallow	HE601843	(Ödeen, et al. 2011)	Hirundo
Passeriformes	Donacobiidae	*Donacobius atricapilla*	black-capped donacobius	HE601848	(Ödeen, et al. 2011)	Donacobiu
Passeriformes	Zosteropidae	*Zosterops lateralis*	silver-eye	HM159125	Hauber & Chong (GenBank 2010)	Zosterops
Passeriformes	Petroicidae	*Petroica rosea*	rose robin	HE601839	(Ödeen, et al. 2011)	PetroicaR

Passeriformes	Acanthisittidae	*Acanthisitta chloris*	rifleman	HM159126	Hauber & Chong (GenBank 2010)	Acanthisi
Passeriformes	Pittidae	*Hydrornis elliotii*	bar-bellied pitta	HE601813	(Ödeen, et al. 2011)	Pitta
Passeriformes	Thamnophilidae	*Myrmeciza hemimelaena*	southern chestnut-tailed antbird	GQ924591	(Seddon, et al. 2010)	Myrmeciza
Passeriformes	Thamnophilidae	*Phlegopsis nigromaculata*	black-spotted bare-eye	GQ924592	(Seddon, et al. 2010)	Phlegopsi
Passeriformes	Pipridae	*Manacus manacus*	white-bearded manakin	AY227182	(Ödeen and Håstad 2003)	Manacus
Passeriformes	Tityridae	*Onychorhynchus coronatus*	Amazonian royal flycatcher	HE601817	(Ödeen, et al. 2011)	Onychorhy
Passeriformes	Tyrannidae	*Myiarchus tyrannulus*	brown-crested flycatcher	AY227183	(Ödeen and Håstad 2003)	Myiarchus
Passeriformes	Tyrannidae	*Camptostoma obsoletum*	southern beardless tyrannulet	HE601814	(Ödeen, et al. 2011)	Camptosto
Passeriformes	Tyrannidae	*Xolmis irupero*	white monjita	HE601815	(Ödeen, et al. 2011)	Xolmis
Passeriformes	Tyrannidae	*Tyrannus savana*	fork-tailed flycatcher	HE601816	(Ödeen, et al. 2011)	Tyrannus
Psittaciformes	Cacatuidae	*Calyptorhynchus latirostris*	Carnaby's black cockatoo	HM150800	(Carvalho, et al. 2011)	Calyptorh
Psittaciformes	Cacatuidae	*Eolophus roseicapilla*	galah	HM150801	(Carvalho, et al. 2011)	Elophus
Psittaciformes	Cacatuidae	*Cacatua galerita*	sulphur-crested cockatoo	HM150802	(Carvalho, et al. 2011)	Cacatua
Psittaciformes	Psittacidae	*Ara macao*	scarlet macaw	HM150792	(Carvalho, et al. 2011)	AraM
Psittaciformes	Psittacidae	*Amazona versicolor*	St. Lucia amazon	HM150793	(Carvalho, et al. 2011)	Amazona
Psittaciformes	Psittacidae	*Ara ararauna*	blue-and-yellow macaw	HM150803	(Carvalho, et al. 2011)	AraA
Psittaciformes	Psittacidae	*Psittacus erithacus*	grey parrot	HM150804	(Carvalho, et al. 2011)	Psittacus
Psittaciformes	Psittacidae	*Ara chloropterus*	red-and-green macaw	HM150805	(Carvalho, et al. 2011)	AraC
Psittaciformes	Psittacidae	*Anodorhynchus hyacinthinus*	hyacinth macaw	HM150806	(Carvalho, et al. 2011)	Anodorhyn
Psittaciformes	Psittacidae	*Platycercus elegans*	crimson rosella	HM150794	(Carvalho, et al. 2011)	Platycerc
Psittaciformes	Psittacidae	*Barnardius zonarius semitorquatus*	Twenty eight parrot	HM150799	(Carvalho, et al. 2011)	Barnardiu
Psittaciformes	Psittacidae	*Melopsittacus undulatus*	budgerigar	Y11787	(Wilkie, et al. 1998)	Melopsitt
Psittaciformes	Strigopidae	*Nestor notabilis*	Kea	HM150807	(Carvalho, et al. 2011)	Nestor

Table S3. Likelihood scores of codon models used for ancestral reconstruction

Model		**lnL**
codon	M7	-865.7970
	M8	-865.7973

Table S4. Maximum likelihood ancestral reconstruction of ancestral passerine/parrot, an passerine SWS1 pigments, with posterior probabilities (numbering according to bovine rhodo

	Passerine/parrot				Passerine			
AA#	codon	PP	AA	PP	codon	PP	AA	PP
72	CTC	1	(L	1.000)	CTC	1	(L	1.000)
73	AAC	1	(N	1.000)	AAC	1	(N	1.000)
74	TAC	1	(Y	1.000)	TAC	1	(Y	1.000)
75	ATC	1	(I	1.000)	ATC	0.998	(I	1.000)
76	CTG	1	(L	1.000)	CTG	1	(L	1.000)
77	GTG	1	(V	1.000)	GTG	1	(V	1.000)
78	AAC	1	(N	1.000)	AAC	1	(N	1.000)
79	ATC	1	(I	1.000)	ATC	0.998	(I	1.000)
80	TCC	1	(S	1.000)	TCC	1	(S	1.000)
81	TTC	1	(F	1.000)	TTC	0.997	(F	0.999)
82	AGC	1	(S	1.000)	AGC	0.999	(S	1.000)
83	GGC	1	(G	1.000)	GGC	1	(G	1.000)
84	TTC	1	(F	1.000)	TTC	0.998	(F	1.000)
85	CTG	1	(L	1.000)	CTG	0.978	(L	1.000)
**86**	**TCC**	**1**	**(S**	**1.000)**	**TGC**	**0.999**	**(C**	**0.999)**
87	TGC	1	(C	1.000)	TGC	1	(C	1.000)
88	ATC	1	(I	1.000)	ATC	0.998	(I	1.000)
89	TTC	1	(F	1.000)	TTC	0.998	(F	1.000)
**90**	**AGC**	**1**	**(S**	**1.000)**	**AGC**	**1**	**(S**	**1.000)**
91	GTC	1	(V	1.000)	GTC	0.999	(V	1.000)
92	TTC	1	(F	1.000)	TTC	0.998	(F	1.000)
93	ACC	1	(T	1.000)	ACC	1	(T	1.000)
94	GTC	1	(V	1.000)	GTC	0.999	(V	1.000)
95	TTC	1	(F	1.000)	TTC	0.998	(F	1.000)
96	GTC	1	(V	1.000)	GTC	0.999	(V	1.000)
97	TCC	1	(S	1.000)	TCC	1	(S	1.000)
98	AGC	1	(S	1.000)	AGC	0.999	(S	1.000)
99	TCC	1	(S	1.000)	TCC	0.832	(S	0.832)
100	CAG	1	(Q	1.000)	CAG	1	(Q	1.000)
101	GGC	1	(G	1.000)	GGA	0.571	(G	1.000)

Supplementary figures

Figure S1. Alignment of SWS1 opsin gene, helix 2 from Landbirds used in ancestral reconstruction, highlighting sites 86, 90 & 93.

```
                                       1
           7       8     8   9  9      0
           23456789012345678901234567890
Alcedo     LNYILVNISFSGFISCIFSVFTVFVSSSQG
Coracias   LNYILVNISFSGFISCIFSVFTVFVSS---
Dendrocop  LNYILVNISFSGFLSCIFSVFTVFVSSSQG
Trogon     LNYILVNISLGGFIFCVFSVFTVFVSS---
Upupa      LNYILVNISFSGFMSCIFSVFTVFVSSSQG
Falco      --YILVNISFSGFISCIFSVF?VFVS----
Acanthisi  LNYILVNISLSGLLCCILCVFLVFVASTQG
Manacus    -NYILVNISFSGFISCIFSVFTV-------
Myrmeciza  LNYILVNISFSGFLCCIFSVFTVF------
Phlegopsi  LNYILVNISFSGFLCCIFSVFTVFV-----
Pitta      LNYILVNISFSGFL?CIFSVFTV-------
Myiarchus  LNYILVNISVSGFMCCIFSVFTVFVSSSQG
Camptosto  LNYILVNISVSGFMCCIFSVFTV-------
Xolmis     LNYILVNISVSGFMCCIFSVFTV-------
Tyrannus   --YILVNISVSGFMCCIFSVFTVFV-----
Onychorhy  LNYILVNISVSGFFCCIFSVFVV-------
Menura     ------NISVSGFFCCIFCVFTVFVSS?QG
Sericulus  LNYILVNISFSGFLCCIFSVFTVFVSSAQG
Chlamyder  LNYILVNISFSGFLCCIFSVFTVFVSSAQG
PomatostT  LNYILVNISFSGFLCCIFSVFTVFVSSAQG
PomatostR  LNYILVNISFSGFLCCIFSVFTVFVSSAQG
Orthonyx   LNYILVNISVSGFFCCIFCVFTVFVSSAQG
Cnemophil  LNYILVNMSISGLMCCIFSVFTVF------
Toxorhamp  LNYILVNISVSGLMCCVFCIFTV-------
Lichenost  LNYILVNISFAGFMCCIFSVFTVFVSSAQG
Acanthorh  LNYILVNISFAGFMCCIFSVFTVFVS----
MalurusA   LNYILVNISFSGFLCCIFCIFTV-------
MalurusE   LNYILVNISFSGFLCCIFCIFTVFVSSSQG
MalurusCy  ------NISFSGFLCCIFCIFTVFVSSSQG
MalurusS   LNYILVNISFSGFLCCIFCIFTVFV-----
MalurusM   LNYILVNISFSGFLCCIFSVFTVFVSSSQG
MalurusL   LNYILVNISFSGFLCCIFSVFTVFVSS---
MalurusCo  LNYILVNISFSGFLCCIFSVFTV-------
MalurusG   --YILVNISFSGFLCCIFSVFTVFV-----
Clytomyia  LNYILVNISFSGFLCCIFSVFTV-------
Amytornis  ---ILVNISFSGFLCCIFSVFTVF------
Gerygone   ------NISFSGFMCCIFSVFTVFVSSAQG
Creadion   ------NISVSGLMCCVFCIFTVFVSSSQG
Rhipidura  LNYILVNISFSGFLCCIFSVFTVFVSSAQG
CorvusC    -NYILVNISFSGFMCCIFSVFTVF?S----
CorvusF    LNYILVNISFSGFLCCIFSVFTVFISSSQG
```

```
                                       1
           7       8     8   9  9      0
           23456789012345678901234567890
Pica       LNYILVNISFSGFLCCIFSVFTVFISSSQG
Cyclarhis  LNYIL?N?SVSGLMCCVFCIFTVFVASSQ-
Coracina   LNYILVNVSFSGFLCCIFSVFTVF------
Oriolus    LNYILVNVSFSGFLCCIFSVFTVFVSSAQG
Dicrurus   LNYILVNISFSGFLCCIFSVFTVFVSSSQG
Cyanocora  --YILVNISFSGFLCCIFSVFTV-------
Ptiloris   LNYILVNISFSGFLCCIFSVFTVFVSSAQG
PetroicaR  ------NISVSGLMCCIFCLFTVFISSSQG
Parus      LNYILVNISVSGLMCCVFCIFTVFVSSSQG
Sitta      LNYILVNISVSGLMCCIFCIFTVFISSSQG
Turdus     LNYILVNISVSGLMCCVFCIFTVFVSSSQG
Sturnus    ------NISVSGLMCCIFCIFTVFVSSSQG
Mimus      ----LVNISVSGLMCCIFCIFTVFVS?SQG
LusciniaS  LNYILVNISVSGLMCCVFCIFTVFVSSSQG
LusciniaC  LNYILVNISVSGLMCCVFCIFTVFVSSSQG
Phyllosco  LNYILVNISVSGLMMCIFCIFTVFVSSSQG
Hirundo    LNYILVNISVSGLMMCIFCIFTVFVSSSQG
Donacobiu  --YILVNISVSGLMMCIFCIFTVFVSSSQG
Leiothrix  ------NISVSGLMMCVFCIFTVFVSSSQG
Zosterops  ------NISVSGLMMCIFCIF?VFVSSSQG
Cinnyris   LNYILVNISVSGLMCCVFCIFTVFVSSSQG
Serinus    LNYILVNISVSGLMCCVFCIFTVFVASSQG
Sturnella  LNYILVNISVSGLMCCVFCIFTVFVASSQG
Anthus     LNYILVNISVSGLMCCVFCIFTVF------
Lonchura   LNYILVNISVAGLMCCVFCIFTVFIASSQG
Amadina    -NYILVNISVSGLMCCVFCIFTVFVASSQG
Taeniopyg  LNYILVNISVSGLMCCVFCIFTVFIASSQG
Euplectes  LNYILVNISVSGLMCCVFCIFTVFVASSQG
Neochmia   LNYILVNISVSGLMCCVFCIFTVFIASSQG
Cacatua    LNYILVNISFCGFLACIFCIFTVFVSSSQG
Eolophus   LNYILVNISFCGFLACIFCIFTVFVSSSQG
Calyptorh  LNYILVNISFCGFLACIFCIFTVFVSSSQG
Psittacus  LNYILVNISFCGFLACIFCIFTVFVSSSQG
AraA       LNYILVNISFCGFLACIFCIFTVFVSSSQG
AraM       LNYILVNISFCGFLACIFCIFTVFVSSSQG
AraC       LNYILVNISFCGFLACIFCIFTVFVSSSQG
Amazona    LNYILVNISFCGFLACIFCIFTVFVSSSQG
Anodorhyn  LNYILVNISFCGFPACIFCIFTVFVSSSQG
Platycerc  LNYILVNISFCGFLACIFCIFTVFVSSSQG
Barnardiu  LNYILVNISFCGFLACIFCIFTVFVSSSQG
Melopsitt  LNYILVNISFCGFLACIICIFTVFVSSSQG
Nestor     LNYILVNISFCGFLACIFCIFTVFVSSSQG
```

Figure S2. Alignment of visual pigment sequences in *C. nuchalis***.** The seven transmembrane domains (TMD) are indicated by gray shading. Key functional sites are highlighted in black. All site numbers are according to corresponding sites in bovine rhodopsin. The sequence of the SWS2 gene is missing 6 amino acids at 5' end. The intron locations of SWS1, as determined by genomic PCR, are at residues G119, R177-Y178, V233 and Q312-F313 (underlined and in bold).

```
                                                                TMD I
SWS1 M-----------------DEDEFYLFKNQSSVGPWDG---PQYHIAPMWAFYLQTIFMGVVFVVGTPLNAIVLIVTVKYK [ 66]
RH1  M--------------NGTEGQDFYVPMSNKTGVVRSPFEYPQYYLAEPWKFSALAAYMFMLILLGFPINFLTLYVTIQHK [ 66]
RH2  M--------------NGTEGINFYVPMSNKTGVVRSPFEYPQYYLAEPWKYRLVCCYIFFLISTGFPINFLTLLVTFKHK [ 66]
LWS  MATWDGAVFAARRRHDDDDTTRDSVFTYTNSNNTRGPFEGPNYHIAPRWVYNLTSLWMIFVVVASVFTNGLVLVATAKFK [ 66]
SWS2 -------??????RDELP?DFYISAALDAPNLTALSPFLVPQTHLGSPGVFRAMAAFMFLLIALGVPVNALTVVCTAKYK [ 66]

           TMD II                                 TMD III
SWS1 KLRQPLNYILVNISFSGFLCCIFSVFTVFVSSAQGYFVFGKHMCALEGFAGATGGLVTGWSLAFLAFERYIVICKPFGNF [146]
RH1  KLRTPLNYILLNLAVANLFMVFGGFTTTMYTSMNGYFVFGVTGCYIEGFFATLGGEIALWSLVVLAIERYVVVCKPMSNF [146]
RH2  KLRQPLNYILVNLAVADLCMACFGFTVTFYTAWNGYFVFGPIGCAVEGFFATLGGQVALWSLVVLAIERYIVICKPMGNF [146]
LWS  KLRHPLNWILVNLAVADLGETVIASTISVVNQIFGYFILGHPMCIIEGYTVSACGITALWSLAIISWERWFVVCKPFGNI [146]
SWS2 KLRSHLNYILVNLAVANLLVVCVGSTTAFYSFSQMYFALGPTACKVEGFAATLGGMVSLWSLAVVAFERFLVICKPLGNF [146]

             TMD IV                                          TMD V
SWS1 RFSSRHALLVVAATWVIGISVAIPPFLGWSRYVPEGLQCSCGPDWYTVGTKYKSEYYTWFLFIFCFIVPLSLIIFSYSQL [226]
RH1  RFGENHAILGVAFSWIMALACAAPPLFGWSRYIPEGMQCSCGIDYYTLKPEVNNESFVIYMFVVHFMIPLLIIFFCYGNL [226]
RH2  RFSASHAMMGIVFTWVMAISCAAPPLFGWSRYIPEGMQCSCGPDYYTHNPDFHNESYVLYMFVIHFIIPVIIIFFSYGRL [226]
LWS  KFDGKLAVAGVLFSWIWSCAWTAPPIFGWSRYWPHGLKTSCGPDVFSGSTDPGVQSYMVVLMVTCCLFPLSVIIFCYLQV [226]
SWS2 TFRGSHAVLGCAITWIFGLIASAPPLFGWSRYIPEGLQCSCGPDWYTTDNKWNNESYVIFLFCFCFGFPLAVIVLSYGRL [226]

                         TMD VI                                    TMD VII
SWS1 LSALRAVAAQQQESATTQKAEREVSRMVVVMVGSFCLCYVPYAALAMYMVNNRDHGLDLRLVTVPAFFSKSACVYNPIIY [306]
RH1  VCTVKEAAAQQQESATTQKAEKEVTRMVIIMVIAFLICWVPYASVAFYIFTNQGSDFGPIFMTIPAFFAKSSAIYNPVIY [306]
RH2  VCKVREAAAQQQESATTQKAEKEVTRMVILMVLGFMLAWTPYAVVAFWIFTNKGADFTATLMAVPAFFSKSSSLYNPIIY [306]
LWS  WLAIRAVAAQQKESESTQKAEKEVSRMVVVMILAYIFCWGPYTFFACFAAANPGYAFHPLTAALPAFFAKSATIYNPIIY [306]
SWS2 LLTLRAVAKQQEQSATTQKAEREVTKMVVVMVLGFLVCWAPYSAFALWVVTHRGRHFDVGLASIPSVFSKASTVYNPVIY [306]

SWS1 CFMNKQFRACIMETVC-GR-PMTDDSEMSSSAQ-RTEVSSVSSSQVSPS* [356]
RH1  IVMNKQFRNCMITTLCCGKNPLGDEDTSAG----KTETSSVSTSQVSPA* [356]
RH2  VLMNKHFRNCMNTTFCCGKNPFGDEDTSSTVSHNKTEVSSVSSSQVSPA* [356]
LWS  VFMNRQFRNCILQLF--GKKV--DDGSEVSTS--RTEVSSVSNSSVSPA* [356]
SWS2 VFMNKQFRSCM?KLVFCGRSPFGDDDDVSGSSQ-ATQVSSVSSSQVSPA* [356]
```

Figure S3. Phylogenetic relationships of the *C. nuchalis* opsin genes with those of other vertebrates. Opsin sequences isolated in this study from the great bowerbird are highlighted in yellow. These were aligned with other sequences obtained from NCBI using ClustalW and subjected to phylogenetic analyses. Maximum likelihood methods were implemented in the program PHYML 3.0 (Guindon and Gascuel 2003; Guindon, et al. 2005), and Bayesian inference in MrBayes 3.2.1(Ronquist and Huelsenbeck 2003), both under the HKY+I+ Γ model, favoured under the Akaike Information Criterion (AIC) using MrModeltest2.3 (Nylander 2008). For ML and NJ analyses, bootstrapping methods (100 replicates) were used to assess the degree of confidence in nodes of the phylogeny (Felsenstein 1985). The Bayesian analysis was run for 3 million generations, sampling every 100, and a 25% burn-in. Shown is the ML bootstrap consensus tree with both ML and NJ support values. An asterix denotes a posterior probability of ≥0.95 in the Bayesian analysis.

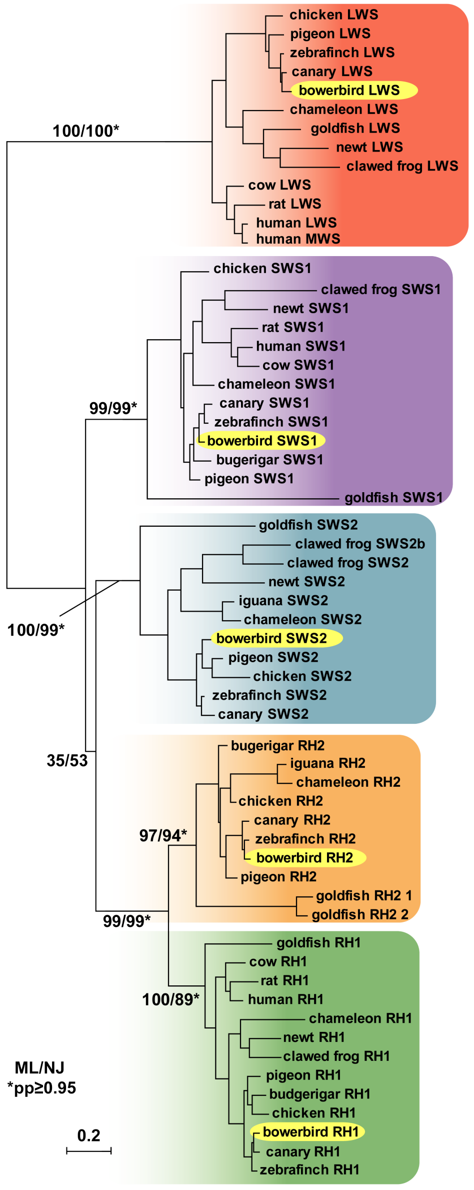

Figure S4. Alternate Landbird topologies used to confirm ancestral sequence reconstruction. Left: original tree based on consensus of current phylogenetics studies (Barker, et al. 2004; Barker, et al. 2002; Beresford, et al. 2005; Driskell, et al. 2011; Gardner, et al. 2010; Hackett, et al. 2008; Irestedt and Ohlson 2008; Johansson, et al. 2008; Jønsson, et al. 2011; Lee, et al. 2012; Suh, et al. 2011; Wang, et al. 2012; Wright, et al. 2008). Right: Secondary tree based on topology of Ödeen et al., (2011). The trees differ in the relationships among higher passerines (Passerida and core-Corvoidea). The reconstructed ancestral nodes are indicated by circles: Passerines (blue), Passerines and Parrots (P + P, green). The corresponding reconstructed fragments are indicated in boxes pointing to their respective nodes. The residue substitutions at sites 86, 90, & 93 are indicated. The reconstructed ancestral nodes are identical in sequence regardless of the topology used. But, the alternate topologies produce slightly dissimilar hypothesis regarding substitutions at site 90 in higher passerines (in red): a single event in the left tree, and two events in the right tree.

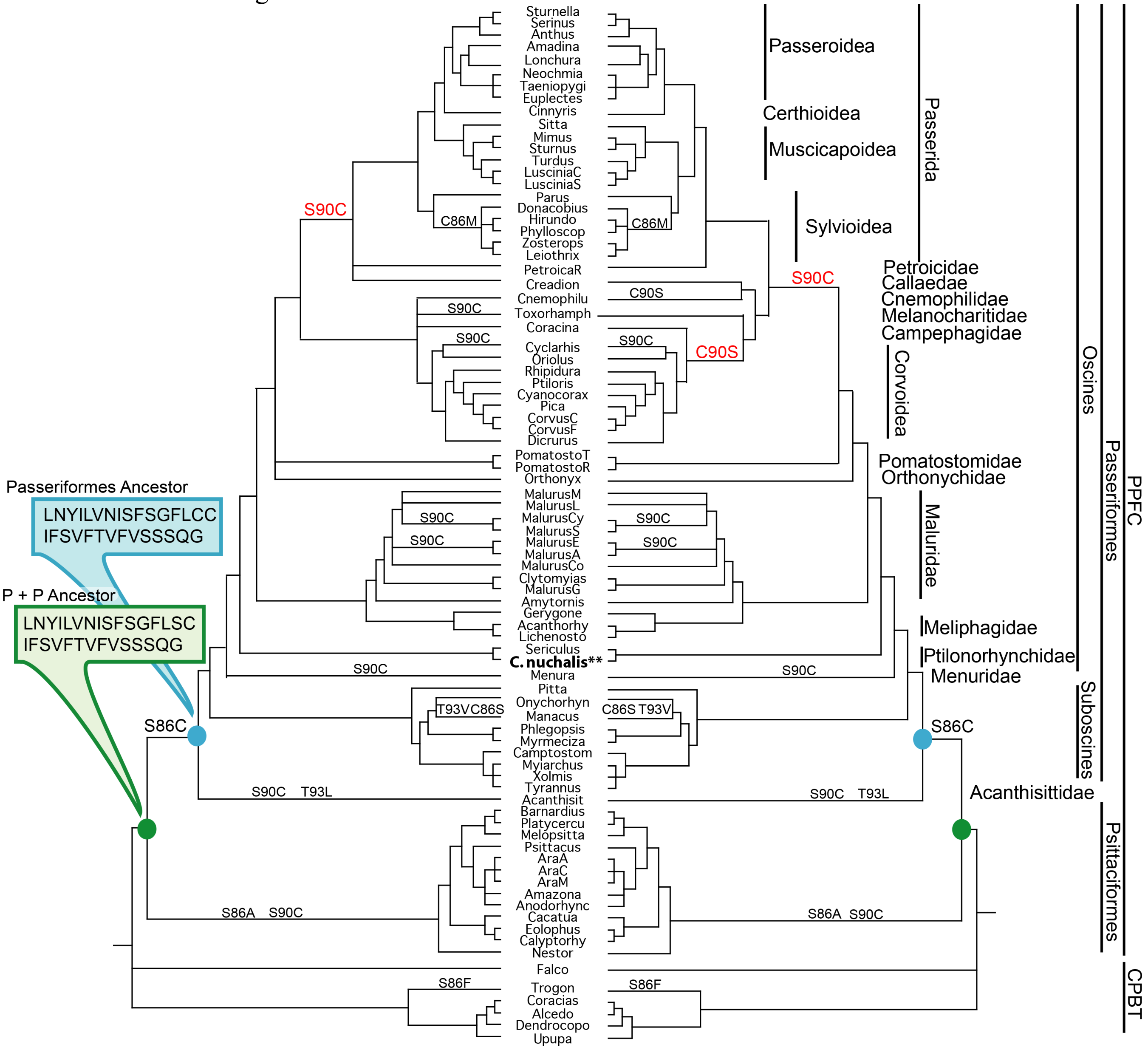

References

Barker F, Cibois A, Schikler P, Feinstein J, Cracraft J 2004. Phylogeny and diversification of the largest avian radiation. Proc Natl Acad Sci U S A 101: 11040-11045.

Barker FK, Barrowclough GF, Groth JG 2002. A phylogenetic hypothesis for passerine birds: taxonomic and biogeographic implications of an analysis of nuclear DNA sequence data. Proc Biol Sci 269: 295-308.

Beresford P, Barker FK, Ryan PG, Crowe TM 2005. African endemics span the tree of songbirds (Passeri): molecular systematics of several evolutionary 'enigmas'. Proc Biol Sci 272: 849-858.

Carvalho LS, Knott B, Berg ML, Bennett AT, Hunt DM 2011. Ultraviolet-sensitive vision in long-lived birds. Proc Biol Sci 278: 107-114.

Das D, Wilkie SE, Hunt DM, Bowmaker JK 1999. Visual pigments and oil droplets in the retina of a passerine bird, the canary Serinus canaria: microspectrophotometry and opsin sequences. Vision Res 39: 2801-2815.

Driskell AC, Norman JA, Pruett-Jones S, Mangall E, Sonsthagen S, Christidis L 2011. A multigene phylogeny examining evolutionary and ecological relationships in the Australo-papuan wrens of the subfamily Malurinae (Aves). Molecular Phylogenetics and Evolution 60: 480-485.

Felsenstein J 1985. Confidence limits on phylogenies: an approach using the bootstrap. Evolution 39: 783-791.

Gardner JL, Trueman JWH, Ebert D, Joseph L, Magrath RD 2010. Phylogeny and evolution of the Meliphagoidea, the largest radiation of Australasian songbirds. Molecular Phylogenetics and Evolution 55: 1087-1102.

Guindon S, Gascuel O 2003. A simple, fast, and accurate algorithm to estimate large phylogenies by maximum likelihood. Syst Biol 52: 696-704.

Guindon S, Lethiec F, Duroux P, Gascuel O 2005. PHYML Online--a web server for fast maximum likelihood-based phylogenetic inference. Nucleic Acids Res 33: W557-559.

Hackett SJ, Kimball RT, Reddy S, Bowie RC, Braun EL, Braun MJ, Chojnowski JL, Cox WA, Han KL, Harshman J, Huddleston CJ, Marks BD, Miglia KJ, Moore WS, Sheldon FH, Steadman DW, Witt CC, Yuri T 2008. A phylogenomic study of birds reveals their evolutionary history. Science 320: 1763-1768.

Irestedt M, Ohlson JI 2008. The division of the major songbird radiation into Passerida and 'core Corvoidea' (Aves: Passeriformes) — the species tree vs. gene trees. Zoologica Scripta 37: 305-313.

Johansson US, Fjeldså J, Bowie RCK 2008. Phylogenetic relationships within Passerida (Aves: Passeriformes): a review and a new molecular phylogeny based on three nuclear intron markers. Molecular Phylogenetics and Evolution 48: 858-876. doi: 10.1016/j.ympev.2008.05.029

Jønsson KA, Fabre P-H, Ricklefs RE, Fjeldså J 2011. Major global radiation of corvoid birds originated in the proto-Papuan archipelago. Proc Natl Acad Sci U S A 108: 2328-2333.

Lee JY, Joseph L, Edwards SV 2012. A species tree for the Australo-Papuan Fairy-wrens and allies (Aves: Maluridae). Syst Biol 61: 253-271.

Nylander J. 2008. MrModeltest v2.3 Program distributed by the author Evolutionary Biology Centre, Uppsala Sweden.

Ödeen A, Håstad O 2003. Complex distribution of avian color vision systems revealed by sequencing the SWS1 opsin from total DNA. Mol Biol Evol 20: 855-861.

Ödeen A, Håstad O 2009. New primers for the avian SWS1 pigment opsin gene reveal new amino acid configurations in spectral sensitivity tuning sites. J Hered 100: 784-789.

Ödeen A, Håstad O, Alström P 2011. Evolution of ultraviolet vision in the largest avian radiation - the passerines. Bmc Evolutionary Biology 11: 313.

Ödeen A, Pruett-Jones S, Driskell AC, Armenta JK, Hastad O 2012. Multiple shifts between violet and ultraviolet vision in a family of passerine birds with associated changes in plumage coloration. Proc Biol Sci 279: 1269-1276.

Ronquist F, Huelsenbeck JP 2003. MrBayes 3: Bayesian phylogenetic inference under mixed models. Bioinformatics 19: 1572-1574.

Seddon N, Tobias JA, Eaton M, Odeen A 2010. Human Vision Can Provide a Valid Proxy for Avian Perception of Sexual Dichromatism. Auk 127: 283-292.

Suh A, Paus M, Kiefmann M, Churakov G, Franke FA, Brosius J, Kriegs JO, Schmitz J 2011. Mesozoic retroposons reveal parrots as the closest living relatives of passerine birds. Nature Communications 2: 443.

Wang N, Braun EL, Kimball RT 2012. Testing hypotheses about the sister group of the passeriformes using an independent 30-locus data set. Mol Biol Evol 29: 737-750.

Wilkie SE, Vissers PM, Das D, Degrip WJ, Bowmaker JK, Hunt DM 1998. The molecular basis for UV vision in birds: spectral characteristics, cDNA sequence and retinal localization of the UV-sensitive visual pigment of the budgerigar (*Melopsittacus undulatus*). Biochemistry Journal 330 (Pt 1): 541-547.

Wright TF, Schirtzinger EE, Matsumoto T, Eberhard JR, Graves GR, Sanchez JJ, Capelli S, Müller H, Scharpegge J, Chambers GK, Fleischer RC 2008. A multilocus molecular phylogeny of the parrots (Psittaciformes): support for a Gondwanan origin during the cretaceous. Mol Biol Evol 25: 2141-2156.

Yokoyama S, Blow NS, Radlwimmer FB 2000. Molecular evolution of color vision of zebra finch. Gene 259: 17-24.
